# An Aeroplysinin-1 Specific Nitrile Hydratase Isolated from the Marine Sponge *Aplysina cavernicola*

**DOI:** 10.3390/md11083046

**Published:** 2013-08-21

**Authors:** Bartosz Lipowicz, Nils Hanekop, Lutz Schmitt, Peter Proksch

**Affiliations:** 1 Institute of Pharmaceutical Biology and Biotechnology, Heinrich-Heine University, Universitaetsstrasse 1, Bldg. 26.23, 40225 Duesseldorf, Germany; E-Mail: lipowicz@uni-duesseldorf.de; 2 Institute of Biochemistry, Heinrich-Heine University, Universitaetsstrasse 1, Bldg. 26.42, 40225 Duesseldorf, Germany; E-Mail: lutz.schmitt@uni-duesseldorf.de

**Keywords:** sponges, *Aplysina cavernicola*, alkaloids, biotransformation, nitrile hydratase

## Abstract

A nitrile hydratase (NHase) that specifically accepts the nitrile aeroplysinin-1 (**1**) as a substrate and converts it into the dienone amide verongiaquinol (**7**) was isolated, partially purified and characterized from the Mediterranean sponge *Aplysina cavernicola*; although it is currently not known whether the enzyme is of sponge origin or produced by its symbiotic microorganisms. The formation of aeroplysinin-1 and of the corresponding dienone amide is part of the chemical defence system of *A. cavernicola*. The latter two compounds that show strong antibiotic activity originate from brominated isoxazoline alkaloids that are thought to protect the sponges from invasion of bacterial pathogens. The sponge was shown to contain at least two NHases as two excised protein bands from a non denaturating Blue Native gel showed nitrile hydratase activity, which was not observed for control samples. The enzymes were shown to be manganese dependent, although cobalt and nickel ions were also able to recover the activity of the nitrile hydratases. The temperature and pH optimum of the studied enzymes were found at 41 °C and pH 7.8. The enzymes showed high substrate specificity towards the physiological substrate aeroplysinin-1 (**1**) since none of the substrate analogues that were prepared either by partial or by total synthesis were converted in an *in vitro* assay. Moreover *de-novo* sequencing by mass spectrometry was employed to obtain information about the primary structure of the studied NHases, which did not reveal any homology to known NHases.

## 1. Introduction

**Figure 1:**
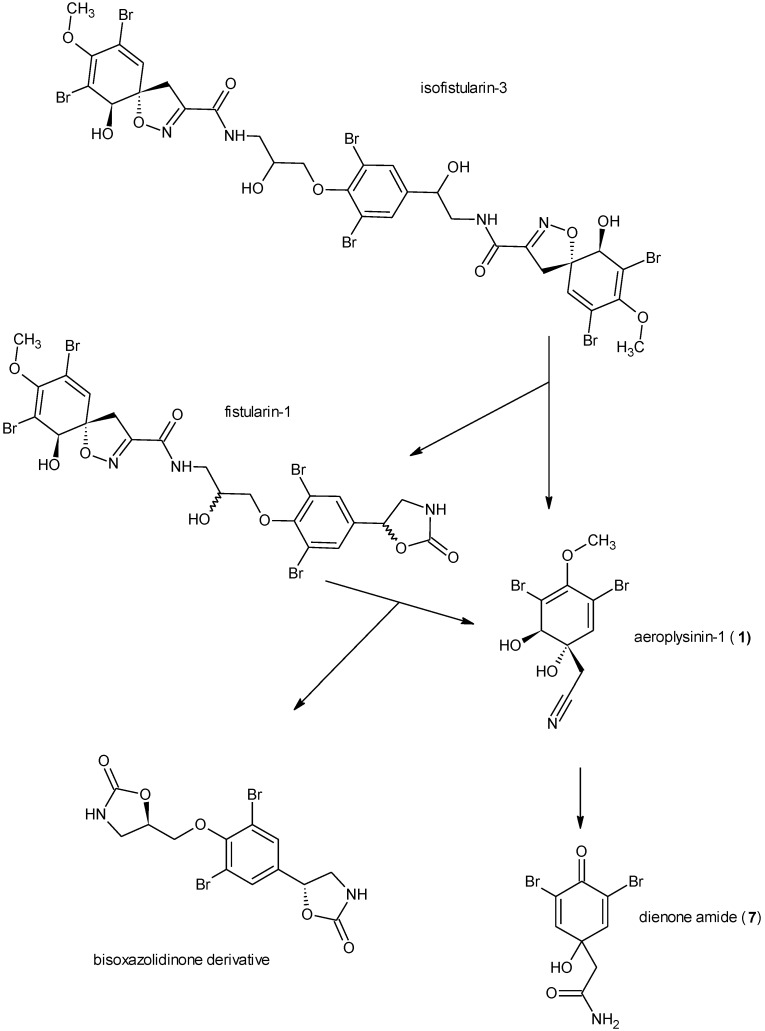
Biotransformation of the isoxazoline alkaloids as described by Teeyapant and Proksch [4] with isofistularin-3 as an example.

Chemical defence of marine organisms may be modulated through (i) pre-formed compounds that are constitutively present, (ii) *de novo* biosynthesis of defensive compounds that are formed following an attack by predators or microorganisms or (iii) biotransformation of defence molecules following disturbance of the cellular compartmentalization [1]. The latter defence strategy is called “activated defence”. Activated defence metabolites originate from inactive or only mildly active precursors by a rapid bioconversion that usually proceeds within seconds following an attack in order to provide fast and efficient protection against intruders [1,2]. In the marine environment, the first example for an activated defence was reported for green algae of the genus *Halimeda* where tissue damage leads to a conversion of the diterpene halimedatetraacetate to the reactive halimedatrial which serves as a defensive metabolite [2]. A further example from algae is known from *Caulerpa taxifolia* where caulerpenyne is transformed to reactive aldehydes including oxytoxins 1 and 2 [3]. Activated defence mechanisms have also been demonstrated for sponges and other marine invertebrates. For sponges, the first example reported was *Aplysina aerophoba* [4]. *A. aerophoba* occurs in the Mediterranean Sea and in the Atlantic Ocean and like other species of this genus, accumulates brominated isoxazoline alkaloids such as the aerophobins, isofistularin-3 or aerothionin that may account for up to 10% of the dry weight [5,6]. These compounds act as feeding deterrents against the marine fish *Blennius sphinx* and are thought to protect the sponges from predators [7]. Upon wounding of the sponge tissue, a rapid conversion of the brominated isoxazoline alkaloids is observed that proceeds through opening of the isoxazoline ring which yields the nitrile aeroplysinin-1 (**1**) (Figure 1) and a carbamic acid derivative (the latter is rapidly cleaved to the corresponding amine and CO_2_ due to the instability of the carbamic acid group). We could previously provide preliminary evidence in favour of the enzymatic nature of this unusual reaction that is so far unprecedented [4,8,9,10]. We could further show that the isoxazoline cleaving enzyme is membrane bound [11]. This wound induced alkaloid bioconversion is not restricted to *A. aerophoba* but is also found in the closely related species *A. cavernicola* that is likewise found in the Mediterranean Sea [7]. In a second enzymatically catalyzed reaction that occurs in *A. aerophoba* and in *A. cavernicola*, the nitrile aeroplysinin-1 (**1**) is further converted to the corresponding dienone amide verongiaquinol (**7**) [4,8]. Both aeroplysinin-1 (**1**) and the dienone amide show strong antibiotic activity against a broad spectrum of gram positive and gram negative marine and terrestrial bacteria and are believed to protect the sponge from infection with microbial pathogens at the site of wounding. The formation of aeroplysinin-1 (**1**) and of the dienone amide thus fulfils the requirements of an activated chemical defence as both bioactive compounds are only formed upon disruption of the cellular compartmentalization of *Aplysina* sponges. 

The transformation of aeroplysinin-1 (**1**) to the dienone amide (**7**) proceeds via hydratation of the nitrile group and is thus likely to be catalysed by a nitrile hydratase. By definition NHases convert aliphatic nitriles into the corresponding amides; however, the recent literature (for review see [12]) uses a wider definition of NHases, which includes enzymes that accept aromatic nitriles as substrates. The physiological roles of known NHases are mostly restricted to the primary metabolism [13,14,15,16,17,18] as microorganisms use the nitrile hydratase/amidase system for the assimilation of ammonia. In a first step, nitriles are converted into their corresponding amides, and in a second step, the amides are hydrolyzed into the corresponding acids and ammonia. NHases are also important for biotechnological applications. More than 100,000 annual tons of acrylamide [19] and several thousand annual tons of nicotinamide are produced with the help of these enzymes [20].

In contrast to bacteria and fungi, there are no reports in the literature [21] on the occurrence of nitrile hydratases in sponges. In this article, we report the partial purification and characterisation of nitrile hydratases isolated from the Mediterranean sponge *A. cavernicola* that convert aeroplysinin-1 (**1**) into the corresponding dienone amide (**7**) *in vitro*, although it is not yet clear whether the enzymes are of sponge origin or produced by its symbiotic microorganisms.

## 2. Results

### 2.1. Partial Purification and Kinetics of the Nitrile Hydratases

**Figure 2:**
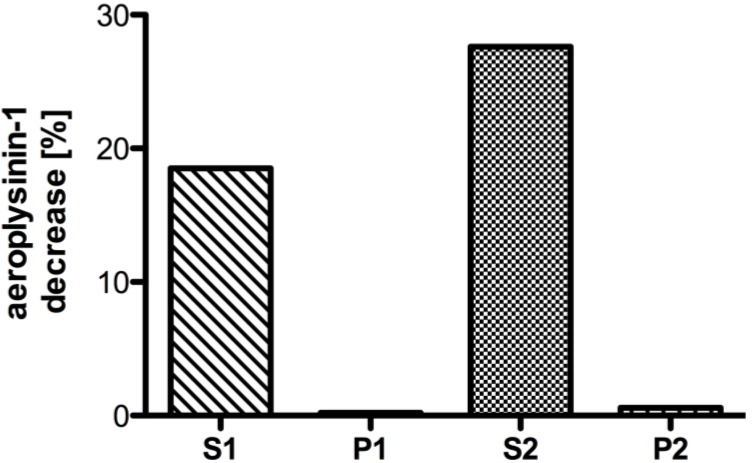
Decrease of the substrate aeroplysinin-1 (in %) relative to a control without enzyme for fractions derived from differential centrifugation at 800 *g* and 100,000 *g*. The supernatant S1 and pellet P1 resulted from a centrifugation step at 800 *g*, while the supernatant S2 and pellet P2 were obtained from a centrifugation step at 100,000 *g*. Activity (in %) is defined as the decrease of substrate relative to a control lacking the enzyme fraction. The activity is calculated for 10 µg of protein. (One representative experiment shown).

After cell lysis by sonification, the enzymes were isolated and partially purified from the protein extract of *A. cavernicola* in four steps. First, the protein extract was subjected to two subsequent centrifugation steps at 800 *g* for 20 min followed by ultracentrifugation of the supernatant of the first centrifugation (S1) at 100,000 *g* for 90 min. Supernatants (S1 and S2) and pellets (P1 and P2) obtained after both centrifugation steps were assayed *in vitro* for enzymatic activity using the physiological substrate aeroplysinin-1 (**1**) and employing a HPLC based activity assay. With the help of this activity assay, both the disappearance of the substrate (**1**) as well as the formation of the product (**7**) could be detected simultaneously by HPLC. Enzyme activity was exclusively confined to the supernatants S1 and S2 indicating that the studied nitrile hydratase is a soluble rather than a membrane bound or membrane-associated enzyme (Figure 2).

Next, the supernatant S2 was subjected to a fractionated ammonium sulphate precipitation, which yielded four fractions. However, when the resulting fractions were desalted by dialysis in lactic acid buffer at pH 3.9, none of them showed measurable enzyme activity in the *in vitro* assay (at pH 7.8).

**Figure 3:**
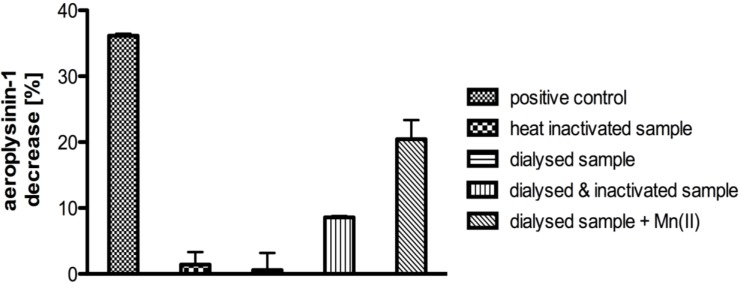
Decrease of the substrate aeroplysinin-1 (in %) relative to a control without enzyme for the supernatant S2 (positive control), a heat inactivated sample, a dialysed sample, a dialysed sample plus a heat inactivated sample and a dialysed sample with 7.5 mM Mn^2+^. Activity (in %) is defined as the decrease of substrate relative to a control without enzyme. The activity is calculated for 10 µg of protein. The experiments were performed in triplicate (*n* = 3) and the error bars are defined as standard deviation.

Addition of an aliquot of heat inactivated (incubation at 95 °C for 20 min) supernatant S2 to the dialyzed protein fraction that had precipitated between 41% and 67% ammonium sulphate was found to restore the enzymatic activity. This indicated the presence of non-proteinaceous low molecular weight cofactors in the protein extract S2, which were lost during ammonium sulphate precipitation and dialysis (Figure 3). As iron or cobalt ions had been reported earlier to act as cofactors of nitrile hydratases [12,13,22], we subsequently analysed the supernatant S2 by flame atomic absorption spectroscopy for the presence of these as well as of other metal ions. Manganese ions showed the highest concentration with 15.6 µg per g of sponge tissue (fresh weight), followed by nickel and cobalt at concentrations of 3.4 and 1.3 µg per g of fresh weight, respectively. Interestingly, iron could not be detected in the supernatant S2. Based on these results, we added manganese ions (final concentration of 7.5 mM) to the dialyzed enzymatically inactive protein fraction that had precipitated between 41% and 67% ammonium sulphate, which likewise restored enzymatic activity (Figure 3). Consequently, subsequent *in vitro* assays were carried out following addition of manganese ions at a final concentration of 7.5 mM if not stated otherwise.

**Figure 4:**
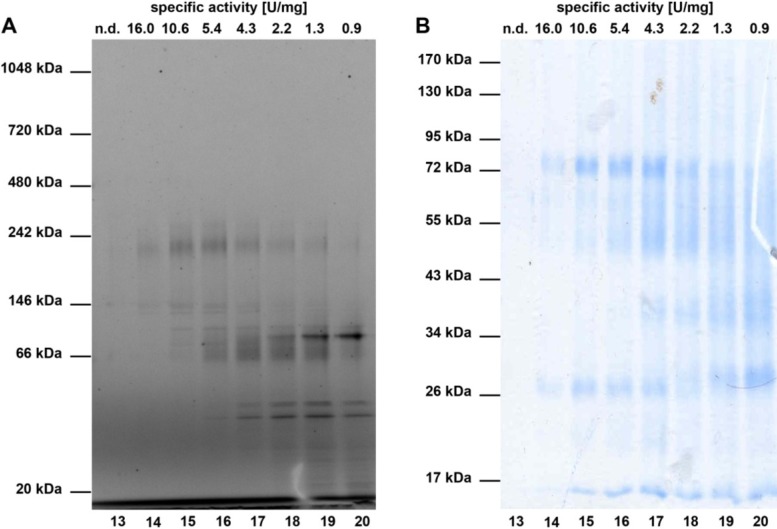
Blue Native-PAGE (**A**) and SDS-PAGE (**B**) analysis of typical size exclusion chromatography fractions. In (**A**), a Coomassie stained NativePAGE 4%–16% Bis-Tris gel of typical SEC fractions is shown. The molecular weights of reference proteins are depicted on the left. (**B**) shows a Coomassie stained 12% SDS-PAGE of the same fractions as in (**A**). The molecular weights of the standard proteins are given next to the gel. Above the gels in (**A**) and (**B**), the specific activity (in U/mg) of the respective fraction with the HPLC based assay (see methods) are shown.

Next, ammonium sulphate precipitation fraction P 67% (precipitating between 41% and 67% ammonium sulphate), which showed the highest enzyme activity after addition of manganese ions, was further separated by ion exchange chromatography at pH 3.9. All fractions were assayed for enzyme activity and additionally subjected to SDS-PAGE. The fractions that showed the highest enzymatic activity and exhibited the least number of protein bands in SDS-PAGE were pooled and further purified via size exclusion chromatography on a Superdex 200 10/300 gel filtration column. Subsequently, the enzymatic activity of the resulting fractions was determined and the samples were analysed by SDS- and Blue Native-PAGE (Figure 4), respectively. Overall, a 52-fold purification of the studied nitrile hydratases compared to the original cell free extract was obtained (Table 1).

**Table 1:** Purification of *A. cavernicola* nitrile hydratases from 10 g of lyophilized sponge tissue.

Purification step	Total protein (mg)	Total activity (U)	Specific activity (U/mg)	Yield (%)
Cell-free extract	114.1	36.1	0.31	100
Ammonium sulphate fractionation	30.9	10.8	0.35	29.9
HiTrap Q Sepharose XL	2.4	1.08	0.45	2.9
Superdex 200 10/300	0.05	0.81	16.2	1.3

**Figure 5:**
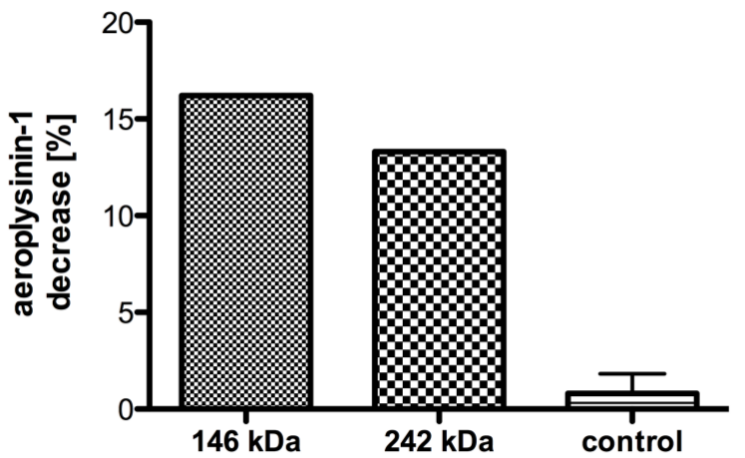
Decrease of the substrate aeroplysinin-1 (in %) relative to a control without enzyme for gel segments excised from the protein bands at 146 and 242 kDa (SEC fraction equivalent to fraction 14 in Figure 4; *n* = 1) in Blue Native-PAGE as well as other parts of the gel (control; *n* = 3). The activity is defined as the decrease of substrate relative to a measurement without gel. The error bars are defined as standard deviation.

The SDS-PAGE of the most active SEC fractions revealed two prominent bands at 26 and 72 kDa (Figure 4B), which might correspond to the α and β-subunits found in bacterial nitrile hydratases [12]. In line with this, the intensity of the 26 and 72 kDa bands diminishes gradually in fractions 14–20, which coincides with the decrease in specific activity. Further analysis of the SEC fractions by non-denaturing Blue Native-PAGE (Figure 4A) showed two prominent bands at 146 and 242 kDa, which might correspond to the nitrile hydratases of *A. cavernicola*. To analyse this in more detail, a SEC fraction with high nitrile hydratase activity (equivalent to fraction 14) was separated by non denaturating Blue Native-PAGE in duplicate. After gel electrophoresis, the gel was cut in two parts. One half (gel A) contained only the sample while the second half (gel B) contained a protein molecular weight standard in addition to the sample. Subsequently, gel B was stained with Coomassie solution according to Dyballa & Metzger [23] whereas gel A was kept overnight in MES buffer at 8 °C. On the following day, the bands at 146 and 242 kDa were excised from gel A (not stained) based at the height of the Coomassie stained protein bands on gel B. Additionally, three arbitrarily chosen gel segments from gel A that did not correspond to either the 146 or the 242 kDa band were excised. All excised gel segments were incubated in a buffer solution pH 7.8 containing 7.5 mM manganese ions. Following addition of aeroplysinin-1 (**1**) and further incubation for 20 min at 21 °C, the enzymatic activity was analysed by HPLC. As depicted in Figure 5, the excised gel segments at 146 and at 242 kDa clearly showed nitrile hydratase activity, which was not observed for the control samples, indicating that the sponge *A. cavernicola* or its symbiotic microorganisms contain at least two nitrile hydratases.

To obtain sequence information on the nitrile hydratases, the 26 and 72 kDa bands in SDS-PAGE were digested with trypsin and the resulting peptides were analysed by mass spectrometry. For the latter *de-novo* sequencing was employed, which derives the peptide sequences solely from the tandem mass spectrum (MS/MS; for details see [24]). Unfortunately, the five peptides (8–20 amino acids in length; see Table 2) of which the sequence could be identified by *de-novo* sequencing did not show any homology to known nitrile hydratases using Blastp (BLASTP 2.2.28+) [25]. However due to the fact that *de-novo* sequencing cannot differentiate leucine and isoleucine residues (due to the identical mass) and has difficulties to distinguish lysine and glutamine residues (very similar mass) it is difficult to identify homologies.

**Figure 6:**
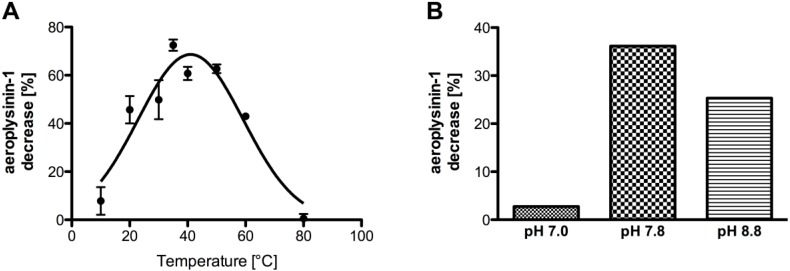
(**A**) Temperature-dependence of the activity of the purified enzyme (SEC fraction 14 or equivalent; *n* = 3). The data were analysed assuming a Gaussian distribution. The activity (in %) is defined as the decrease of substrate relative to a control without protein. The activity is calculated for 0.1 µg of protein. The error bars are defined as standard deviation. (**B**) Influence of the pH on the activity of the nitrile hydratases in the supernatant S2. The activity (in %) is defined as the decrease of substrate relative to a control without protein. The activity is calculated for 10 µg of protein.

Further characterisation of the studied nitrile hydratases revealed a temperature optimum at 41 °C (Figure 6A). The pH optimum of the enzyme was detected at pH 7.8 (Figure 6B), which is similar to the pH of ambient sea water. Due to the low solubility of aeroplysinin-1 (**1**) in aqueous methanolic solution that was used for the enzymatic *in vitro* enzyme assay Michaelis-Menten kinetics did not give satisfactory results both for *K_m_* and for *V*_max_ as saturation of the enzyme was not reached before the substrate aeroplysinin-1 precipitated at concentrations higher than 36 mM.

**Table 2:** Peptide masses obtained from *de-novo* sequencing by tandem mass spectrometry. The table shows the molecular mass ([M + H]^+^), the charge (*z*) and the mass-charge ratio (*m/z*) of the peptide fragments obtained from tandem mass spectrometry (MS/MS) along with the sequence of the fragments. The amino acid sequences are given in single letter code. Sequences in parenthesis denote amino acids of which the order could not be determined experimentally [e.g., (FN) is either FN or NF]. Furthermore it is not possible to differentiate L and I by mass spectrometry due to the identical mass, therefore the peptide fragments contain only L. Similarly, it is very difficult to distinguish Q and K by mass spectrometry, therefore within a peptide Q is reported as K would have been a cleavage site for trypsin and thus can only be found at the end of the peptide (assuming complete cleavage).

Molecular weight ([M + H]^+^)	Charge (z)	Mass-charge ratio (*m/z*)	Sequence
914.46	++	457.73	LSSEFGFK
960.56	++	480.78	FVTPLDLR
1187.64	++	594.37	WDETVVALVR
1937.92	+++	646.64	(FN)FDLTHQQQLDYLR
2269.15	+++	757.05	DLPASANDLPYFLLHAQLDR

### 2.2. Influence of Different Metal Ions on the Enzymatic Activity

**Figure 7:**
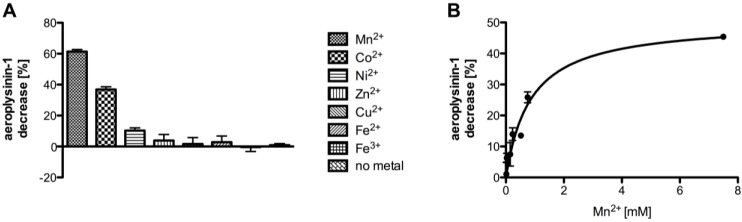
(**A**) Influence of selected metal ions (each at a concentration of 7.5 mM) on the activity of the purified nitrile hydratases (SEC fraction 14 or equivalent; *n* = 3). (**B**) Influence of different concentrations of Mn^2+^ on the activity of the purified enzyme (SEC fraction 14 or equivalent; *n* = 3). The data were analysed assuming a one site binding model, which resulted in an apparent affinity constant of 0.856 ± 0.228 mM for Mn^2+^ ions. The activity is defined as the decrease of substrate (in %) relative to a control without protein. The activity is calculated per 0.1 µg of protein. The error bars are defined as standard deviation.

As manganese ions were capable of restoring the enzymatic activity of dialysed protein fractions originating from fractionated ammonium sulphate precipitation, we subsequently analysed the effects of cobalt, copper, iron, manganese, nickel and zinc ions on the activity of the nitrile hydratases of *A. cavernicola* using fractions from size exclusion chromatography. Figure 7A shows the influence of the different metal ions on the enzymatic activity at a concentration of 7.5 mM. Zinc copper and iron had—despite the high concentrations used—no effect whereas manganese cobalt and nickel restored the enzymatic activity of the nitrile hydratases. From the latter three metal ions manganese showed the most pronounced effect and resulted in a 60% conversion of the substrate aeroplysinin-1 relative to a control without enzyme. Cobalt and nickel—when added at equimolar concentrations compared to manganese—were clearly less effective and restored the enzyme activity only to 37% and 10% respectively. 

Since manganese had the strongest effect on the enzymatic activity we also analysed the influence of different manganese concentrations (Figure 7B). The addition of manganese ions at a final concentration of 30 µM to the SEC fractions restored the enzyme activity to 6% whereas no effect was seen at a manganese concentration of 15 µM. A maximum increase of enzyme activity (45%) was found following the addition of 7.5 mM manganese ions to the assay. Assuming a one site binding model for the binding of the Mn^2+^ ions, this resulted in an apparent affinity constant of the nitrile hydratases of 0.856 ± 0.228 mM.

### 2.3. Substrate Specificity

**Figure 8:**
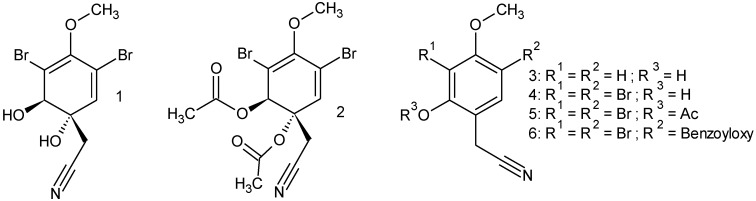
Aeroplysinin-1 and its derivatives.

To test the substrate specificity of the studied nitrile hydratases, we prepared (either by total synthesis or by derivatization of compound **1**) several derivatives of the physiological substrate aeroplysinin-1 (**1**) as no analogues were commercially available that shared important structural features with aeroplysinin-1 such as a brominated, hydroxylated and methoxylated six-membered ring with an acetonitrile side chain in position 1 (Figure 8).

**Figure 9:**
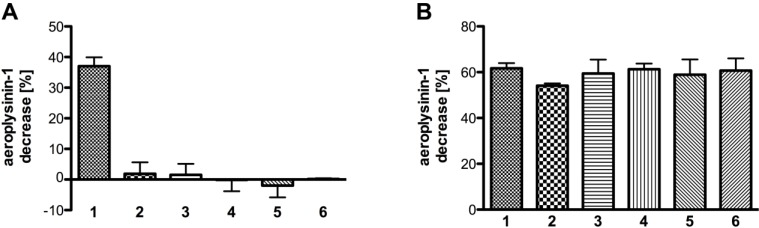
(**A**) The turnover of aeroplysinin-1 and selected derivatives by the purified enzyme (SEC fraction 14 or equivalent; *n* = 3); (**B**) Competition between aeroplysinin-1 and its derivatives (at equimolar concentrations with SEC fraction 14 or equivalent; *n* = 3). The activity (in %) is defined as the decrease of substrate relative to a control without enzyme. The activity is calculated per 0.1 µg of protein. The error bars are defined as standard deviation.

None of the tested analogues (**2**–**6**) except the natural substrate aeroplysinin-1 (**1**) was converted by the enzyme (Figure 9A) An equimolar concentration of the derivatives **2** to **6** in addition to aeroplysinin-1 had no effect on the turnover of the natural substrate (Figure 9B) suggesting that the analogues do not bind to the enzyme.

From the tested analogues compound **2** was obtained by acetylation of aeroplysinin-1 (**1**) and differed from the latter only by the presence of acetyl functions instead of hydroxyl groups at positions C-1 and C-2. The importance of the hydroxyl groups in the structural framework of aeroyplysinin-1 is highlighted by the fact that the diacetylated compound **2** is not accepted as a substrate by the isolated nitrile hydratases even though the acetonitrile side chain is still present in the molecule. During the partial hydrolysis of aeroplysinin-1 (**1**) by the studied nitrile hydratases, the hydroxyl group at position 2 is eliminated leading to a shift in the double bond system of the six-membered ring of the product **7**. The presence of a bulky acetate group in this position apparently prevents compound **2** from being accepted as a substrate. Next to compound **2**, the analogues **3** and **4** showed the highest structural similarity with **1** except that both **3** and **4** are aromatic and lack the hydroxyl group at position C-1. As for compound **2**, no enzymatically catalyzed conversion was observed for the latter substrate analogues thus highlighting the pronounced substrate specificity of the studied nitrile hydratases.

## 3. Discussion

In this study, we have partially purified and characterized for the first time nitrile hydratases (NHase) from the Mediterranean sponge *Aplysina cavernicola* that convert the sponge derived nitrile aeroplysinin-1 (**1**) into the corresponding dienone amide (**7**). We thus confirmed on a biochemical level the wound induced alkaloid biotransformation that had been claimed earlier for this sponge [4,8,9]. Nitrile hydratase activity has been demonstrated in the past mainly for bacteria [12,13,26], yeasts [27,28] and moulds [29]. Recent molecular studies have, however, shown that nitrile hydratase genes are also found in other eukaryotic supergroups, but so far not in metazoa [21,30]. The first report of a eukaryote featuring nitrile hydratase encoding genes was from the choanoflagellate *Monosiga brevicollis* [30]. Choanoflagellates are the closest sister group of the metazoa and therefore, the closest non-metazoan relative of the porifera as sponges represent the oldest animal phylum [31]. The isolation of nitrile hydratases from *A. cavernicola* would be the first example for the detection of this group of enzymes in an animal provided that the sponge and not symbiotic bacteria are the source of the enzyme. Nevertheless based on the fact that nitrile hydratases are so far unreported for sponges, it is more likely to assume that microbial symbionts of the sponge that may account for up to 40% of the sponge biomass [32,33] are the true sources of the studied enzymes. Support for this hypothesis comes from a recent paper [34] that reports the presence of bacterial halogenase genes in symbionts of *A. aerophoba*, the sister species of *A. cavernicola* that likewise occurs in the Mediterranean Sea. These halogenases are possibly involved in the bromination of the isoxazoline alkaloids which are precursors to aeroplysinin-1 (**1**). 

The results obtained by Blue Native-PAGE in combination with activity assays (Figure 4 and Figure 5) indicated the presence of at least two nitrile hydratases in *A. cavernicola*, which have molecular weights of approximately 146 kDa and 242 kDa, respectively. Unfortunately, attempts to obtain sequence information from mass spectrometry of trypsin treated protein samples were unsuccessful, because the peptides identified by *de-novo* sequencing did not show any homology to known nitrile hydratases. However, the estimated sizes of both enzymes are in the range of known nitrile hydratases, which have molecular weights, from 54 [35] to 500 kDa [36]. The presence of two rather than one nitrile hydratase in the same organism as found in this study for *A. cavernicola* has also been reported for other organisms such as for the gram-positive bacterium *Rhodococcus rhodochrous*, which is commercially employed as a biocatalyst for the industrial production of acrylamide [37]. 

Interestingly, the characterisation of the partially purified nitrile hydratases from *A. cavernicola* showed that the addition of manganese ions, which to a certain extent can be replaced by cobalt and nickel ions (Figure 7A), is necessary for enzymatic activity. In line with this, manganese ions showed the highest concentration (238 nM) in the enzymatically active supernatant S2 followed by nickel (48 nM) and cobalt (19 nM). Taken together, these results suggest that manganese ions might be bound as cofactors in the active centre of the nitrile hydratases from *A. cavernicola*. This is in clear contrast to the nitrile hydratases discovered so far that either harbour cobalt or iron ions in the catalytic centre [12,13,22]. The only known exception from the strict dependence of nitrile hydratases on cobalt and iron ions in the catalytic centre is an enzyme isolated from *Rhodococcus* sp. RHA1 that has been reported to contain one cobalt, two copper and one zinc ion per functional enzyme [38]. Nevertheless, we have to admit that the concentrations of manganese ions (and also of cobalt and nickel ions) necessary to recover the activity of the nitrile hydratases from *A. cavernicola* are very high (in the high µM to low mM range) and will probably not be reached *in vivo*. However, similar results were obtained for the expression of recombinant nitrile hydratases in *Escherichia coli* [39]. Here, the addition of high micromolar concentrations of cobalt ions (the studied enzyme belongs to group of cobalt-dependent nitrile hydratases) to the medium or to the cell extract were necessary to obtain active proteins. A possible explanation for the observed phenomenon might be the activator proteins found in the nitrile hydratase/amidase operons of several bacteria (for review see [12]), although their function is not fully understood. Nevertheless, the activator proteins are thought to modify the active site of the NHase [40,41], facilitate the transport of the metal ion [42] or the binding of the metal ion to the subunits of the NHase [43]. In a nitrile hydratase from *R. rhodochrous* J1, for example, an activator protein—NhlE—was necessary for the activation of the nitrile hydratase [44]. NhlE forms a complex with the α subunit of the nitrile hydratase (NhlA), which is required for the post-translational modification of specific cysteine residues in the active centre of the alpha-subunit and for the incorporation of cobalt ions. Furthermore, the maturation mediator NhlAE catalysed the exchange of its α-subunits with apo NhlAB, which is inactive, resulting in the mature (active) nitrile hydratase in a process called self-subunit swapping [44]. In light of the latter, a possible explanation for the high manganese concentrations required for the reactivation of the nitrile hydratases from *A. cavernicola* lies in the fact that *in vivo* additional protein(s) are involved in the maturation of the protein, and here especially, the incorporation of manganese ions. If these proteins are missing (as in our study), it seems plausible that the (re-) incorporation of the metal ions into the protein requires higher metal ion concentrations, which is different from the *in vivo* situation.

Further characterisation of the nitrile hydratases from *A. cavernicola* revealed a temperature and pH optimum at 41 °C and at pH 7.8, respectively. The pH optimum coincides with the pH of ambient sea water that usually varies from pH 7.8 to 8.2 [45] whereas water temperatures in the Mediterranean Sea may fluctuate from 13 °C in the winter to 24 °C in the summer (Marine Station Hydra, Elba, personal communication). Similar pH and temperature optima as found in this study have also been reported for other nitrile hydratases [26,36,46,47].

The nitrile hydratases from *A. cavernicola* seem to be highly specific for their physiological substrate aeroplysinin-1 (**1**) as five different structural analogues were not converted to the corresponding amide, which suggests a very narrow substrate spectrum of the enzyme. The high substrate specificity of the nitrile hydratases from *A. cavernicola* is unusual for this group of enzymes as most other known nitrile hydratases (for an exception see [29]) accept a wide range of substrates which makes them interesting tools for biotechnology [20,37,48,49].

Usually kinetic parameters of bacterial NHases towards the most common nitriles such as acrylonitrile and cyanopyridine show *K_m_* values in the mM range (1–20 mM). In our study, it was not possible to determine the *K_m_* and *V*_max_ values reliably for the studied nitrile hydratases due to an impaired solubility of the physiological substrate aeroplysinin-1 (**1**) that precipitated in the aqueous/methanolic enzyme assay at concentrations >36 mM. At this concentration, substrate saturation of the enzymes had not been reached. Nevertheless the physiological concentration of brominated isoxazoline alkaloids that are transformed in a likewise enzymatically catalyzed reaction yielding aeroplysinin-1 (**1**) [11] amounts to roughly 35 mmol per g of fresh sponge tissue. Local alkaloid concentrations may even be considerably higher than this average value since isoxazoline alkaloids in *Aplysina* species are known to be preferentially localized in specialized spherolous cells at the surface of the sponge and around the excurrent channels [50,51]. Thus, isoxazoline concentrations, and in turn, local aeroplysinin-1 concentrations in spherolous cells can be expected to be considerably higher than the average value of 35 mmol per g fresh sponge tissue. The isoxazoline converting enzyme that cleaves alkaloid precursors such as the aerophobins and converts them to aeroplysinin-1 (**1**) has been shown to be a membrane bound enzyme [11]. The nitrile hydratases that are the subject of the present study are soluble enzymes. It may nevertheless be hypothesized that *in situ* the nitrile hydratases are spatially associated with the isoxazoline cleaving enzyme as this would minimize diffusion of the substrate aeroplysinin-1 and allow the nitrile hydratases to work at maximum speed.

One important difference of the nitrile hydratases from *A. cavernicola* compared to nitrile hydratases reported in other organisms derives from the fact that the sponge enzymes not only convert the nitrile group into the corresponding amide but (1) also eliminate the hydroxyl group at the C-2 of the substrate which results in the formation of the double bond Δ^2,3^ in the product **7** and (2) they demethylate the methoxy substituent thereby giving rise to the oxo function at C-4 in the resulting dienone amide (**7**). Preliminary data indicate that the hydroxyl group at C-2 of the substrate aeroplysinin-1 (**1**) is used for an intramolecular addition of water to the nitrile function of aroplysinin-1. When the enzymatic *in vitro* assay with aeroplysinin-1 (**1**) as substrate was carried out in a buffer solution where H_2_^16^O had been replaced by H_2_^18^O, no increase in the molecular weight of the dienone amide by two mass units was detected using mass spectrometry as would have been expected in the case of an extramolecular water addition [52].

The main difference of the studied nitrile hydratases from *A. cavernicola* compared to other nitrile hydratases is, however, functional. Microorganisms use nitrile hydratases in combination with amidases usually for the assimilation of ammonia and hence, for their primary metabolism [13,14,15,16,17,18]. In the case of *A. cavernicola*, the nitrile hydratases clearly have a role in the secondary metabolism of the sponge as part of an enzymatic machinery that generates highly toxic and antibiotically active metabolites from isoxazoline precursors that act as pro-drugs in a wound activated chemical defence reaction [7,8,53,54]. Due to its strong antibiotic activity, the dienone amide (**7**) has even been proposed for use as an antibiotic for mariculture as it rivals chloramphenicol with regard to its efficiency in decreasing mortality of cultured callop (*Pecten maximus*) larvae due to bacterial infections [55]. Thus, it can be expected that the wound induced formation of aeroplysinin-1 (**1**) and of the dienone amide (**7**) that occurs at the site of wounding of sponge tissue following mechanical damage or injury [9] will protect the sponge from invading microorganisms. The studied enzymes thus play an important role in the chemical defence of *A. cavernicola* and protect the sponge from microbial hazards.

## 4. Experimental Section

### 4.1. Origin of Sponge Material

The sponge was collected near the island of Elba (Mediterranean Sea) at a depth of 40 m next to “Capo di Fonza”. After sampling, the fresh sponge was directly frozen in liquid nitrogen and subsequently kept frozen at −20 °C until lyophilisation. The lyophilized sponge was then ground in an ice-cold mortar until a fine powder was reached. The powder was stored at −80 °C until it was used for extraction of proteins.

### 4.2. Protein Extraction

An aliquot of 2.5 g of lyophilized and ground sponge tissue was suspended in 20 mL of 50 mM MES buffer at pH 5.8 and extracted for 45 min in an ice-cold ultrasonic bath. After incubation, the samples were centrifuged (Heraeus Multifuge X1R, Highconic II rotor, Thermo Scientific, Langenselbold, Germany) for 20 min at 800× *g* and 4 °C. The supernatant was transferred into fresh centrifuge tubes while the pellet (P1) was resuspended in 5 mL of buffer (see above) and centrifuged for 15 min at 800× *g* and 4 °C. Both supernatants (S1) were combined and submitted to ultracentrifugation (Sorvall Discovery, Ti 60 rotor Beckmann, Krefeld, Germany) for 90 min at 100,000× *g* and 4 °C. This supernatant (S2) was used in further purification steps.

### 4.3. Ammonium Sulphate Fractionation

S2 was submitted to fractionated ammonium sulphate precipitation. Proteins precipitating between 41% and 67% saturation at 4 °C were collected by centrifugation (Biofuge pico, Heraeus, Hanau, Germany) for 5 min at 13,000 rpm. The pellet was re-suspended in 1 mL of 50 mM lactic acid at pH 3.9 and dialyzed against the respective buffer. After dialysis, the suspension was centrifuged for 5 min at 16,000 *g* to remove insoluble proteins from the supernatant (P2 dialyzed).

### 4.4. Ion Exchange Chromatography

The dialyzed protein fraction was applied to a 1 mL HiTrap Q Sepharose XL column (GE Healthcare, Freiburg, Germany) that had been equilibrated with 50 mM lactic acid at pH 3.9 (buffer A). The column was washed with the respective buffer until unbound protein had been washed off followed by a gradient elution for 24 min, starting with 0% buffer B (buffer A + 1 M NaCl) and ending with 100% buffer B. The flow rate was set to 1 mL/min. The ion exchange chromatography was performed on ÄKTAprime plus FPLC system that recorded the absorption at 280 nm.

### 4.5. Size Exclusion Chromatography

For gel filtration, a Superdex 200 10/300 gel filtration column (GE Healthcare, Freiburg, Germany) was employed using the same Äkta-FPLC system as described for ion exchange chromatography. Prior to usage, the column was equilibrated with 2 column volumes of 50 mM MES pH 5.8 containing 0.1 M NaCl. Active enzyme fractions from ion exchange chromatography that showed the least complex band pattern in SDS-PAGE were pooled and concentrated via an Amicon Ultra-15 (Millipore, Schwalbach, Germany; MWCO = 3 kDa) to a total volume of approximately 500–600 μL. Subsequently, the concentrated fractions were injected onto the equilibrated Superdex 200 column at a flow rate of 0.5 mL/min and the separation of the proteins was monitored at 280 nm. 

### 4.6. Protein Analysis

Protein concentration was determined using a Bradford assay [56]. SDS-gel electrophoresis was performed according to Laemmli [57] using a 12% SDS-gel. After electrophoresis, the SDS-gels were stained with Coomassie brilliant blue G250 [23]. Blue Native-PAGE was performed with a NativePAGE Novex 4%–16% Bis-Tris Gel (Life Technologies, Darmstadt, Germany) according to Schägger and Jagow [58].

### 4.7. Activity Assay

An aliquot of 5 μL of each enzyme fraction obtained during the different purification steps was tested for activity. The total assay volume was 10 μL and contained 5 μL enzyme solution, 3 μL of 25 mM manganese(II) (final concentration in the assay 7.5 mM), 0.6 μL of 1.5 M Tris-HCl buffer at pH 8.8, 0.6 μL of 1 M HEPES buffer at pH 7 and 0.8 μL of 29 mM aeroplysinin-1 (**1**) dissolved in methanol. The assay mixture was incubated for 20 min at 21 °C. The reaction was stopped by freezing the sample in liquid nitrogen. Prior to HPLC analysis, each sample was thawed, diluted in 60 μL methanol and directly injected into a HPLC system (Waters 510 pump, Waters 717plus autosampler, TechLab K3 column oven, Knauer Eurospher 100-C18, 5 μm, 125 × 4 mM i.d. column, Waters 996 photodiode array detector, isocratic elution, solvent system methanol:0.1% trifluoroacetic acid (1:2 v/v), flow rate 1.5 mL/min, detection at 280 nm). Activity was defined as the decrease of the substrate aeroplysinin-1 (**1**) in % compared to a negative control that was identical to the assay described above but lacked the enzyme solution.

### 4.8. Analysis of Metals in the Supernatant S2

Metal analysis of the supernatant S2 was carried out using a fast sequential atomic absorption spectrometer AA240FS (Varian, Palo Alto, USA). The single-jet spectrometer used was operated with an acetylene air flame (acetylene 13.5 L/min, air 2.0 L/min). As a control the respective buffer was analysed.

### 4.9. Activity Assay with Excised Protein Bands from Blue Native-PAGE

An active protein fraction eluting from the size exclusion column that showed two major bands in the SDS-PAGE at 26 and 72 kDa was separated via Blue Native-PAGE as described above. The fraction was spotted on two lanes. Next to one lane, a protein molecular weight standard (NativeMark Unstained Protein Standard, Life Technologies, Darmstadt, Germany) was loaded. After electrophoresis, the gel was cut vertically, separating the two sample lanes (gel A contained only the protein samples, while gel B contained the protein sample and the molecular weight standard). Gel B was stained with a Coomassie staining solution overnight whereas gel A was kept in 50 mM MES buffer of pH 5.8 at 8 °C. Following staining with Coomassie, both gels were compared and at the height of the stained *A. cavernicola* proteins from gel B gel pieces were excised from gel A, which corresponded to the two major protein bands at 146 kDa and 242 kDa. Three further gel pieces from gel A that did not correspond to a protein lane were likewise cut out and subsequently treated like the protein bands. All gel pieces were put into separate buffer solutions containing 500 μL of 50 mM MES-Puffer at pH 5.8, 300 μL of 25 mM manganese(II), 75 μL of 1.5 M Tris-HCl buffer at pH 8.8 and 75 μL of 1 M HEPES buffer at pH 7 followed by an incubation for 30 min at 21 °C. After this pre-incubation, 50 μL of an aeroplysinin-1 solution (900 μM, dissolved in methanol) was added and all samples were incubated for an additional 20 min at 21 °C. The activity assay was stopped by freezing the samples in liquid nitrogen. For analysis, the samples were thawed and directly injected onto the HPLC system. The chromatograms were recorded at 280 nm and activity was defined as the decrease of the substrate in % compared to control samples that were prepared as described above but lacking gel pieces. 

### 4.10. Influence of Different Metal Ions on the Enzymatic Activity

Several metals were evaluated for their effect on the enzyme activity. The assay was performed as described under “activity assay”, but instead of manganese, the assay contained various other metal ions at a concentration of 7.5 mM. Samples containing aeroplysinin-1 and 7.5 mM Mn^2+^ served as a positive control. Negative controls were prepared by replacing the enzyme solution in the assay by 50 mM MES buffer at pH 5.8 (*n* = 3). 

### 4.11. Substrate Specificity

Substrate specificity was analyzed using several synthetic or semi-synthetic derivatives of aeroplysinin-1. The assay was the same as described under “activity assay”, but aeroplysinin-1 was replaced by the analogues **2** to **6** (Figure 8). As several substrate analogues were rather lipophilic, all compounds were dissolved in an equal volume of methanol and DMSO resulting in a final substrate concentration of 29 mM. Samples containing aeroplysinin-1 (**1**) served as a positive control, whereas negative controls contained 50 mM MES buffer at pH 5.8 instead of enzyme solution (*n* = 3).

### 4.12. Inhibition Assay

An inhibition assay was performed to determine the structural requirements for possible substrates. The assay was performed as described under “substrate specificity”. Instead of using 0.8 μL of 29 mM aeroplysinin-1 or its analogues, 0.8 μL of an equimolar mixture of aeroplysinin-1 and one of the derivatives was used. Samples containing only aeroplysinin-1 served as a positive control, whereas negative controls contained of 50 mM MES buffer at pH 5.8 instead of the enzyme solution (*n* = 3).

### 4.13. Enzyme Kinetics

Kinetic measurements were carried out with aeroplysinin-1 as substrate and three different metal ions including manganese, cobalt and nickel. The measurements were performed as described under “activity assay” and included final substrate concentrations ranging from 0.009 to 36 mM in the assay (*n* = 3).

In a second assay, the manganese concentration was varied from 15 μM to 7.5 mM at a constant aeroplysinin-1 concentration of 29 mM (as described under *activity assay*; *n* = 3). 

### 4.14. Restoring Enzymatic Activity

The supernatant S2 from ultracentrifugation was incubated for 20 min at 95 °C in a heating block (Heating block UBD, Grant, Shepreth, England) followed by centrifugation for 5 min at 16,000 *g* (Biofuge pico, Heraeus, Hanau, Germany). For the activity assay, 30 μL of this inactivated sample was added to 30 μL of a dialyzed protein fraction obtained after dialysis of the fraction that had precipitated between 41% and 67% ammonium sulphate. An aliquot of 0.6 μL of 1.5 M Tris-HCl buffer at pH 8.8, 0.6 μL of 1 M HEPES buffer at pH 7 and 0.8 μL of 29 mM aeroplysinin-1 was added and the sample was incubated for 20 min at room temperature. The reaction was stopped by freezing samples in liquid nitrogen. Prior to the measurement, each sample was thawed and directly injected into the HPLC system as described under “activity assay”. Chromatograms were recorded at 280 nm and activity was defined as the decrease of the substrate in % compared to a negative control consisting of 50 mM MES buffer at pH 5.8 instead of the dialyzed protein sample (*n* = 3). 

### 4.15. Synthesis

Synthesis was performed according to Farkas *et al*. (1971) [59] and Andersen and Faulkner (1975) [60]. Oxidation with lead(IV)tetraacetate was performed according to Lee and Su (2007) [61]. Impurity with benzylchloride led to benzoyloxylated compounds.

#### 4.15.1. Diacetylaeroplysinin-1 (**2**)

^1^H NMR (500 MHz, CDCl_3_) δ 6.53 (s, 1H), 6.20 (s, 1H), 3.75 (s, 3H), 3.06 (dd, *J* = 16.91, 40.87 Hz, 2H), 2.21 (s, 3H), 2.07 (s, 3H); ESI-MS *m/z* 446.6 [M + Na]^+^.

#### 4.15.2. 2-Hydroxy-4-methoxyphenylacetonitril (**3**)

^1^H NMR (500 MHz, acetone-*d*_6_) δ 7.21 (d, *J* = 8.40 Hz, 1H), 6.51 (d, *J* = 2.50 Hz, 1H), 6.47 (dd, *J* = 2.52, 8.40 Hz, 1H), 3.73 (s, 3H), 3.69 (s, 2H); EI-MS *m/z* 163.

#### 4.15.3. 3,5-Dibromo-2-hydroxy-4-methoxyphenylacetonitril (**4**)

^1^H NMR (500 MHz, methanol-*d*_4_) δ 7.51 (s, 1H), 3.84 (s, 3H), 3.80 (s, 2H); ESI-MS *m/z* 320 [M − H]^−^.

#### 4.15.4. 3,5-Dibromo-2-acetyl-4-methoxyphenylacetonitril (**5**)

^1^H NMR (500 MHz, methanol-*d*_4_) δ 7.73 (s, 1H), 3.89 (s, 3H), 3.83 (s, 2H), 2.40 (s, 3H); EI-MS *m/z* 363.

#### 4.15.5. 3,5-Dibromo-2-benzoyloxy-4-methoxyphenylacetonitril (**6**)

^1^H NMR (500 MHz, dichloromethane-*d*_2_) δ 8.25 (dd, *J* = 1.23, 8.32 Hz, 2H), 7.73 (t, *J* = 7.48 Hz, 1H), 7.73 (s, 1H), 7.59 (t, *J* = 7.84 Hz, 2H), 3.92 (s, 3H), 3.67 (s, 2H); 2.40 (s, 3H); EI-MS *m/z* 425.

### 4.16. Mass Spectrometric Analysis of Peptide Fragments

The MS analysis was performed at the BMFZ, Heinrich-Heine University (Duesseldorf, Germany). For MS analysis, the sample was separated by SDS-PAGE (see above) and stained with Coomassie according to Dyballa & Metzger [23]. Subsequently, the bands of interest were excised from the SDS-PAGE and the protein was digested with proteomics grade trypsin (Sigma-Aldrich, Taufkirchen, Germany) for 12–16 h at 37 °C. The resulting peptides were eluted from the gel and the pooled extracts were dried in a vacuum centrifuge. Before MS analysis, the peptides were desalted using a C18 Stage-tip (Proxeon, Thermo Scientific, Langenselbold, Germany). The ESI-MS analyses were performed with an ESI Qq-TOF instrument equipped with a nanospray source (Q-STAR XL, Applied Biosystems, Darmstadt, Germany). The peptide sequences were determined by *de-novo* sequencing. 

## 5. Conclusions

In conclusion, two nitrile hydratases from the Mediterranean sponge *Aplysina cavernicola* were partially purified that are highly specific for their substrate aeroplysinin-1 (**1**) as none of the substrate derivatives analyzed in this study was converted by the enzymes into the corresponding amide. In contrast to nitrile hydratases reported so far, which reveal a strict dependence on either iron or cobalt ions as cofactors, the *A. cavernicola* enzymes seem to possess manganese ions in the active centre. Furthermore *de-novo* sequencing of the enzymes by mass spectrometry did not show any homology to known nitrile hydratases. In light of the different reactions catalyzed by “classical” nitrile hydratases and the *A. cavernicola* enzymes the latter is not surprising as the sponge enzymes in addition to converting a nitrile group into the corresponding amide also eliminate the hydroxyl group at the C-2 of the substrate and demethylate its methoxy substituent at the C-4.
